# Correlation of CAG repeat length between the maternal and paternal allele of the Huntingtin gene: evidence for assortative mating

**DOI:** 10.1186/1744-9081-7-45

**Published:** 2011-10-18

**Authors:** Peg Nopoulos, Eric A Epping, Tom Wassink, Bradley L Schlaggar, Joel Perlmutter

**Affiliations:** 1 Department of Psychiatry, University of Iowa Carver College of Medicine, Iowa City, Iowa, USA; 2 Department of Pediatrics, University of Iowa Carver College of Medicine, Iowa City, Iowa, USA; 3 Department of Neurology, University of Iowa Carver College of Medicine, Iowa City, Iowa, USA; 4 Department of Radiology, Washington University School of Medicine, St. Louis, MO, USA; 5 Department of Pediatrics, Washington University School of Medicine, St. Louis, MO, USA; 6 Department of Anatomy & Neurobiology, Washington University School of Medicine, St. Louis, MO, USA; 7 Department of Neurology, Washington University School of Medicine, St. Louis, MO, USA; 8 Department of Occupational Therapy, Washington University School of Medicine, St. Louis, MO, USA

## Abstract

Triplet repeats contribute to normal variation in behavioral traits and when expanded, cause brain disorders. While Huntington's Disease is known to be caused by a CAG triplet repeat in the gene Huntingtin, the effect of CAG repeats on brain function below disease threshold has not been studied. The current study shows a significant correlation between the CAG repeat length of the maternal and paternal allele in the Huntingtin gene among healthy subjects, suggesting assortative mating.

## Introduction

Huntington's Disease (HD) is a neurodegenerative disorder caused by a triplet repeat expansion of the gene Huntingtin (*HTT*, OMIM 613004). Triplet repeats are an example of simple sequence repeats (SSRs) which are scattered throughout the genome and can increase or decrease in length between generations. Triplet repeats can be referred to as 'dynamic mutations' and they make up a large class of genomic variants that contribute to a wide variety of disorders, mostly affecting the brain [1]. More importantly, there is increasing evidence that dynamic mutations serve important functions (namely regulation of gene expression) and may play a substantial role in modulating brain development and brain function [1-9]. For instance, SSRs are particularly abundant in genes involved in brain development and have been shown to contribute to normal variation in behavioral traits in animals and humans [3]. These SSRs therefore may have provided the variability needed for the changes of brain development and function in the primate lineage leading to human evolution [4].

In sexually reproducing populations, mating does not occur randomly, but in relationship to certain characteristics - either with a positive correlation (a male pairs with a similar female) or a negative correlation (a male pairs with a dissimilar female). This phenomenon, termed assortative mating, has been widely reported in humans with positive correlations for characteristics such as intelligence [10,11], body size [12-15] education [16], personality characteristics [17-19] and mental disorders [20,21]. A recent review outlined a number of human behaviors that are associated with SSRs including anxiety related traits, novelty seeking behavior, cognitive function, and altruism [3]. Therefore, behaviors modified by SSRs may undergo assortative mating, as shown for the allelic variation of the dopamine receptor D4 (DRD4) gene, an SSR associated with novelty seeking behavior [22].

A better understanding of the function of *HTT *below disease threshold may be important for understanding the disease process of HD. For instance, if there is phenotypic variation in brain structure or function associated with *HTT *CAG length below disease threshold, it may help to define a possible spectrum of phenotype ranging from normal to pathologic. This phenotypic spectrum extends the concept of disease pathology beyond the classic dichotomous categorization between normal and diseased brain. Furthermore, some literature supports the notion of important relationships between the normal HTT allele and the expanded allele, manifesting as differences in disease expression [23].

Although *HTT *has not directly been associated with variance in behavior, it is critical for brain development [24,25] and therefore may be associated with variance in brain structure and function. We sought to evaluate the possibility of assortative mating in a group of subjects recruited from the community and with no family history of Huntington's.

## Methods

As a control group for a study on children at risk for Huntington's Disease, healthy children are recruited from the surrounding community of our hospital via advertising. Through a screening interview with parents, children are excluded if they have a history of significant medical neurologic, or psychiatric history. All participants signed informed consent prior to enrolling in the protocol, which was approved by the local Investigational Review Board (IRB). Participants ages 6-12 years signed both an assent form (language geared toward children) and the consent form.

Several children who participated were siblings and to avoid the confound of genetic relatedness, in the cases in which there were 1 or more siblings, the participants were randomly deleted and only 1 child from each family was included. The demographics of the group included 47 healthy children, including 31 girls and 16 boys, ranging from 6-18 years of age. A total of 40 of the 47 children were Caucasian (85% of the sample), 4 were African American (8.6%) and 3 were multiracial (6.4%).

Each child provided either blood or saliva for genetic analysis. All analyses were done through the University of Iowa Molecular Diagnostics Laboratory. Size of the CAG repeat region of *HTT *on chromosome 4p16.3 was determined with PCR analysis of genomic DNA. PCR primers that exclude the adjacent polymorphic CCG tract were used to amplify the CAG region. A second set of primers that includes the CCG polymorphism is routinely used to assist in differentiating two alleles with an identical CAG repeat number. The CAG repeat length for each subject is determined by comparing the PCR products to sizing standards. By convention, the longest allele is designated as Allele1 and the shorter allele as Allele2. Parent DNA was not available to determine maternal or paternal transmission of each allele.

### Statistical analysis

Normality of distribution of CAG lengths of both alleles was tested using the Shapiro-Wilk test. If either allele was found to be non-normally distributed, then non-parametric analysis was used (Spearman Correlation) to assess the association between length of Allele1 and length of Allele2.

## Results

Distribution of CAG repeat lengths were not normally distributed. For Allele1, the range was from 15 to 30 with mean of 20.20, s.d. of 3.88. Shapiro-Wilk statistic was significant (0.873, p < 0.0001) indicating non-normality of distribution. For Allele2, the range was 4 to 29, mean of 17.29 and s.d. of 3.67. Shapiro-Wilk statistic was significant (0.848, p < 0.0001) again indicating a non-normal distribution.

**Figure 1:**
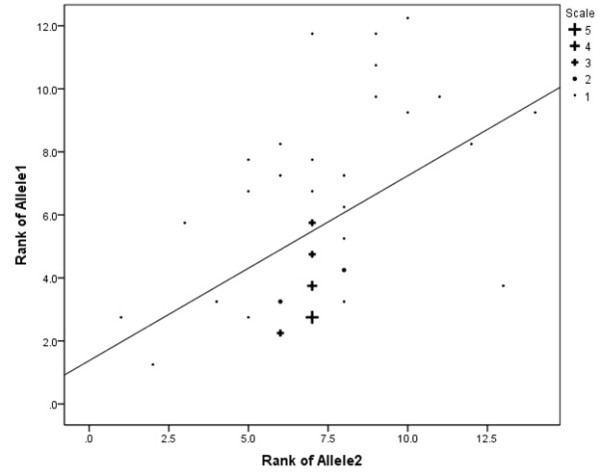
**Relationship between CAG repeat length of Allele1 and CAG repeat length of Allele2**.

The Spearman correlation between Allele1 and Allele2 was highly significant at r = 0.511, p = 0.0002. This observation confirms that longer Allele1 lengths are positively associated with longer Allele2 lengths. Figure 1 shows the scatter plot of the data with regression line displayed.

## Discussion

This simple analysis of a unique data set shows evidence that there is assortative mating in regard to CAG length of *HTT*. That is, the length of CAG repeat in the maternal allele of *HTT *strongly correlates with the length of the CAG repeat of the paternal allele of *HTT*, suggesting that the male and female pair mated based on the common feature of having a similar genotype (length of CAG repeat).

One interpretation of these findings would be that variability of CAG length is manifest by variation in phenotype of brain structure and function. This notion supports a report in which measures of mitochondrial energy metabolism (ATP/ADP) directly correlated to *HTT *CAG repeat lengths below disease threshold [26]. Although in that study the genetic variation was associated with metabolic phenotypic variation, brain structure/function phenotypic variation also may be associated with CAG repeat length in *HTT*. As is seen with other SSRs, variation in multiple types of behavior and cognitive functions have been shown to be associated with variations in repeat sizes of these genetic elements. With expansion of *HTT *CAG repeat length beyond 36 repeats, disease is manifested and the brain region most heavily affected is that of the basal ganglia. Basal ganglia circuits include widespread connections from and to multiple cortical regions, including frontal lobes. These frontal circuits influence numerous complex functions including cognitive and personality traits [27-32]. Potential influence of CAG repeat length on these basal ganglia frontal circuits could influence behaviors that provide the basis for assortative mating.

An alternative explanation for the current findings could be that a post-meiotic recombination between CAG repeat domains of the two *HTT *alleles 'equilibrate' the 2 allele sizes, thereby producing a correlation between the sizes of the *HTT *alleles [33]. To distinguish whether the mechanism responsible for the reported correlation is assortative mating or post-meiotic recombination, future studies will need to analyze parental DNA along with the proband's DNA.

Racial or ethnic relationships within the sample is an important consideration in the current study since there are differences in the distribution of normal *HTT *allele sizes in different ethnic groups [34-38]. Furthermore, different haplotypes associated with different distribution of the normal *HTT *allele sizes may influence the prevalence of HD in certain regions of the world [39,40]. Therefore, individuals choosing mates based on race or ethnicity may explain our current findings of relationships consistent with assortative mating. However, this explanation requires that a substantial number of subjects represent more than one racial or ethnic group. In the current sample, the vast majority (85%) are Caucasian with a small numbers of African Americans (n = 4) or multiracial subjects (n = 3). Moreover, if the correlation between the ranks of Allele1 and Allele2 are calculated within the 40 Caucasians, the relationship remains significant (Spearman's r = 0.408, p = 0.009). Thus, it seems unlikely that the current findings represent assortative mating based on ethnic group. Yet, recent reports of distribution of normal *HTT *allele sizes suggest that the prevalence of modifier genes may be different even *among sub-groups *of Caucasians [40]. Again, this explanation requires multiple subjects within several discreet sub-groups within this sample of 40 Caucasians which, although possible, seems less likely. Thus, assortative mating based not on ethnic group but on some other human feature remains a viable explanation for the findings reported here. Nevertheless, given the preliminary nature of the findings, follow-up in larger samples and further exploration of the functions of the variance of normal CAG length in *HTT *are warranted.

## Competing interests

The authors declare that they have no competing interests.

## Authors' contributions

PN secured funding, completed the analysis, and drafted the manuscript. EA provided feedback and revisions to manuscript. TW provided feedback and revisions to manuscript. BS provided feedback and revisions to manuscript. JP provided feedback and revisions to manuscript. All authors read and approved the final manuscript.
